# Structural transitions of sword bean canavalin in response to different salt concentrations

**DOI:** 10.1016/j.heliyon.2019.e03037

**Published:** 2019-12-18

**Authors:** Kaho Nishizawa, Yasuhiro Arii

**Affiliations:** a Department of Food Science and Nutrition, School of Human Environmental Sciences, Mukogawa Women's University, Nishinomiya, Hyogo, 663-8558, Japan; b Research Institute for Nutrition Sciences, Mukogawa Women's University, Nishinomiya, Hyogo, 663-8558, Japan

**Keywords:** Analytical chemistry, Material science of foods, Food chemistry, Plant biology, Proteins

## Abstract

Canavalin is the major storage protein in sword beans (*Canavalia gladiata*) and belongs to the 7S seed globulin class. Canavalin solubility can be reversibly altered via the addition of MgCl_2_ and CaCl_2_ at different concentrations; specifically, it is insoluble at lower concentrations and soluble at higher concentrations. However, the addition of NaCl does not induce the insolubilization of canavalin. In this study, it was determined that the addition of NaCl causes the nearly complete solubilization of MgCl_2_-precipitated canavalin in the presence of high concentrations. Moreover, using gel filtration we examined the quaternary structures of soluble canavalin in the bean extract and in the presence of high-concentration salts. Results indicated that canavalin was present in the monomer form within crude extracts with distilled water. Alternatively, we identified trimeric soluble canavalin in the presence of high concentrations of NaCl or MgCl_2_. Our study revealed that the quaternary structures of sword bean soluble proteins differ in crude extract compared to those in high-concentration salt solutions. The three-dimensional structure of β-conglycinin, which is a typical 7S-seed globulin in soybean, has a trimer form in the presence of high concentrations of NaCl. However, it remains unclear whether β-conglycinin is present as trimers in soybean seed. Our findings serve as an important reference to analyze 7S globulin characteristics.

## 1 Introduction

The sword bean (*Canavalia gladiata*) is an edible leguminous plant that originated either from southern Asia or Africa (Purseglove, 1968). Sword bean seeds are highly nutritious and contain ~26% protein (Vadivel and Janardhanan, 2005). Considering their agronomical and nutritional features, sword beans are expected to be ideal for use in processed foods as a source of protein. The major protein is canavalin, which belongs to the 7S seed globulin, or vicilin, class with a molecular weight of 47.6 kDa (Sumner et al., 1983).

In our previous study, we established a method to extract the proteins from dried sword bean seeds in distilled water (Nishizawa et al., 2016). Further, its solubility can be reversibly altered via the addition of Mg^2+^ and Ca^2+^ at different concentrations (Nishizawa and Arii, 2016). Moreover, canavalin that is extracted in distilled water is soluble and becomes precipitated following the addition of low concentrations of divalent cations; however, this effect is lost in the presence of higher concentrations of divalent cations. Precipitated canavalin (MgCl_2_-precipitated canavalin) is reversibly resolubilized in the presence of high concentrations of divalent cations, but is not solubilized in distilled water. In addition, NaCl can maintain canavalin in the soluble form in sword bean extracts, regardless of the NaCl concentration (0–400 mM) (Nishizawa and Arii, 2016). The variable solubility of canavalin is an interesting physicochemical property. However, it is unclear whether the addition of NaCl can solubilize MgCl_2_-precipitated canavalin and whether the structures of these soluble proteins differ.

In this study, NaCl was added to MgCl_2_-precipitated canavalin to investigate the ability of NaCl to solubilize MgCl_2_-precipitated canavalin. In addition, the quaternary structures of canavalin were investigated under different conditions using gel filtration. The findings from this study have the potential to serve as an important reference for the analysis of sword bean protein characteristics and 7S globulin characteristics.

## 2 Materials and methods

### 2.1 Materials

White sword beans were purchased from Morika Kometen (Nara, Japan), and general chemical reagents were purchased from Wako Pure Chemical Industries (Osaka, Japan). The molecular weight standard kits (LMW and HMW calibration kits) were purchased from GE Healthcare UK Ltd (Little Chalfont, England).

### 2.2 Preparation of the sword bean extract

The sword bean extract was prepared according to previously described methods (Nishizawa et al., 2016). Dried sword beans were soaked in 10 volumes (v/w) of distilled water at 20 °C for 18 h. The soaked beans were then ground on ice for 5 min in 8 volumes (v/w) of distilled water using a hand blender (CSB-77JBSTRW, Cuisinart, Stamford, CT, USA). The suspension was sieved through a cotton cloth. The extract was centrifuged at 9,100 × *g* for 10 min at 4 °C, and the supernatant was used in the analyses.

### 2.3 Analysis of sword bean proteins

The solubility of sword bean proteins was analyzed using previously described methods (Nishizawa et al., 2016; Nishizawa and Arii, 2016) with specific modifications. Briefly, 150 mM MgCl_2_ or 2 M NaCl (0.1 mL) was added to 9 volumes of sample extract (0.9 mL) and incubated at 25 °C for 15 min and on iced for 5 min. Samples were then separated into supernatant and precipitate via centrifugation at 9,100 *× g* at 4 °C for 20 min. The precipitate (prepared by adding MgCl_2_ to the sword bean extract) was suspended in 60 mM MgCl_2_ or 200 mM NaCl. The mixtures were again separated into the supernatant and precipitate by centrifugation at 9,100 *× g* at 4 °C for 20 min. The precipitates were dissolved in a volume of 8 M urea in a volume equal to that of the salt-added extract (1.0 mL). Sword bean proteins were separated by gel filtration chromatography and analyzed using sodium dodecyl sulfate polyacrylamide gel electrophoresis (SDS-PAGE).

### 2.4 SDS-PAGE

Samples (90 μL) were mixed with 0.33 volumes (30 μL) of SDS sample buffer (0.25 M tris-HCl [pH 7.0], 4% SDS, 5% 2-mercaptoethanol, and 40% glycerol) and incubated at 100 °C for 5 min. SDS-PAGE was conducted with 10% polyacrylamide gels at a constant current of 12.5 mA for 2.5 h, according to the standard method described by Laemmli (1970). Proteins were stained with 0.25% Coomassie Brilliant Blue R-250. The molecular weight standard was purchased from Life Technologies (Tokyo, Japan).

### 2.5 Determination of the residual canavalin ratio

Residual canavalin in the supernatant was quantified using previously described methods (Nishizawa and Arii, 2016). The intensity of canavalin bands on the SDS-polyacrylamide gel was quantified by ImageJ (National Institutes of Health, Bethesda, MD) (Abràmoff et al., 2004). The residual canavalin ratio was expressed as the percentage of band intensity of the NaCl-added samples to that of the samples with distilled water. Data were expressed as means ± standard deviations of three independent replicates.

### 2.6 Gel filtration chromatography

Sword bean proteins were separated using a gel filtration column (HiPrep 16/60 Sephacryl S-200 High Resolution, GE Healthcare UK Ltd, England) with a bed volume of 120 mL in distilled water containing 200 mM NaCl or 60 mM MgCl_2_ at a flow rate of 0.5 mL/min. Samples were injected into the column in volumes of 2.0 mL. Eluted samples were collected in volumes of 1.5 mL. The molecular weight standard was prepared by mixing the LMW calibration kit with aldose from the HMW calibration kit. The standard contained aprotinin, ribonuclease A, carbonic anhydrase, ovalbumin, conalbumin, and aldolase with average molecular masses of 6,500, 13,700, 29,000, 44,000, 75,000, and 158,000 Da, respectively. The collected samples were assayed using Bradford dye reagent (Bio-Rad Laboratories Inc., CA, USA) according to the commercial protocol. Absorbance was measured at 595 nm.

## 3 Results

### 3.1 Effects of NaCl on canavalin solubility

**Figure 1:**
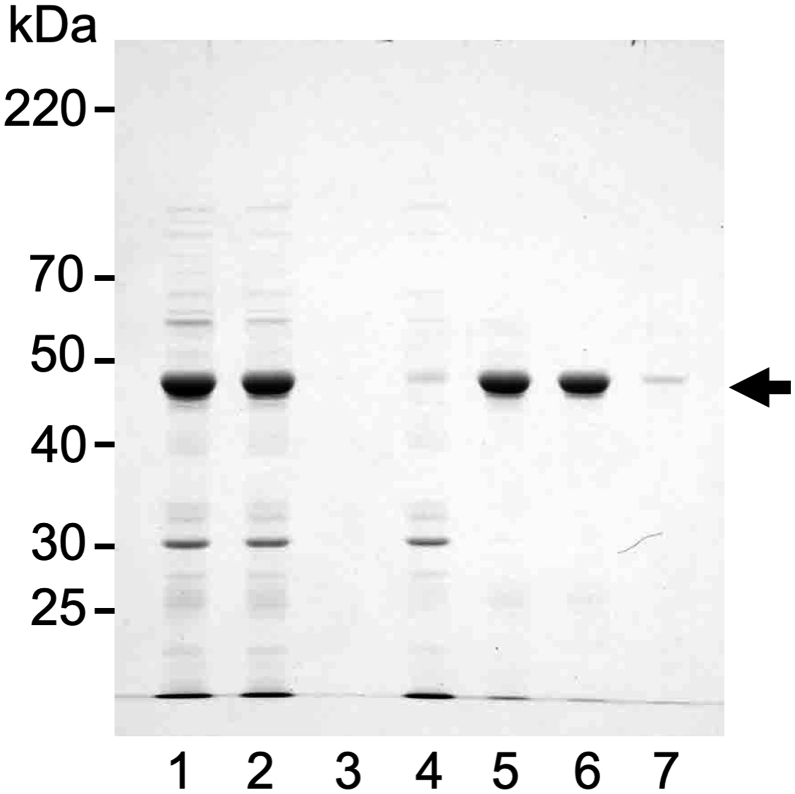
Effect of NaCl on canavalin solubility. NaCl was added to the sword bean extract (lane 1) at a final concentration of 200 mM. The NaCl mixture was separated into the supernatant (lane 2) and precipitate (lane 3) by centrifugation. MgCl_2_ was also added to the sword bean extract at a final concentration of 15 mM. The MgCl_2_ mixture was separated into the supernatant (lane 4) and precipitate (lane 5) by centrifugation. The precipitate from the MgCl_2_ mixture was suspended in 200 mM NaCl. The suspension was again separated into the supernatant (lane 6) and precipitate (lane 7) by centrifugation.

To investigate the effect of NaCl on MgCl_2_-precipitated canavalin, the MgCl_2_-precipitated canavalin was suspended in a high concentration of NaCl (Figure 1). Canavalin was observed in the supernatant (lane 2) but not in the precipitate (lane 3). These results indicated that canavalin was soluble when the concentration of NaCl was high. In contrast, when MgCl_2_ was added to the sword bean extract at a final concentration of 15 mM, most canavalin was observed in the precipitate (lane 5) but not in the supernatant (lane 4) when the concentration of MgCl_2_ was low. Furthermore, the MgCl_2_-precipitated canavalin (lane 5) was suspended in 200 mM NaCl. In the suspension, most canavalin was observed in the supernatant (lane 6) rather than the precipitate (lane 7). The results indicated that the MgCl_2_-precipitated canavalin was solubilized via the addition of 200 mM NaCl.

### 3.2 NaCl concentration-dependent changes in canavalin solubility

**Figure 2:**
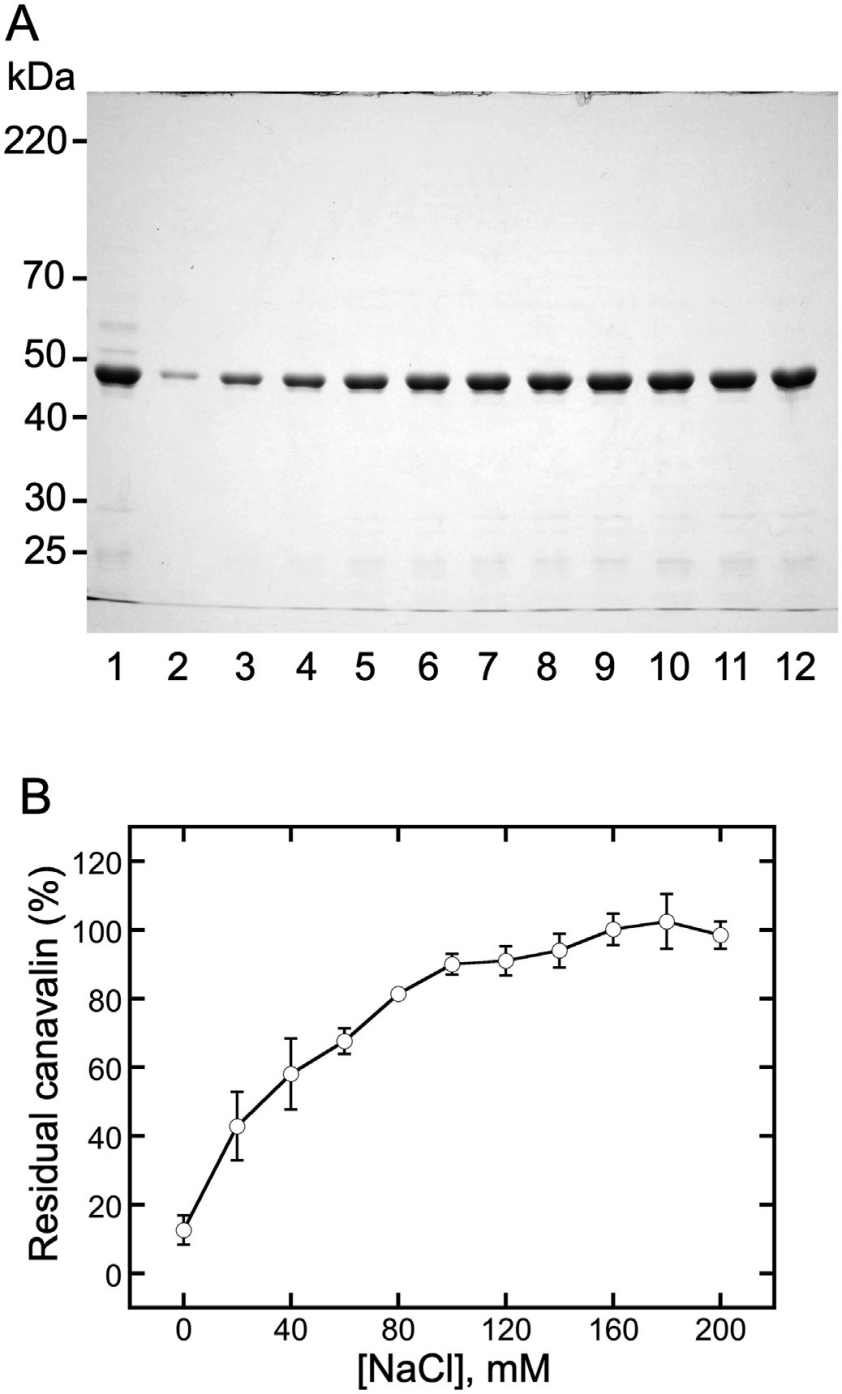
Effect of NaCl concentration on canavalin solubility. (A) Canavalin in the sword bean extract was precipitated via the addition of MgCl_2_ at a final concentration of 15 mM and then suspended in 8 M urea (lane 1), distilled water (lane 2), or NaCl at concentrations of 20 mM (lane 3), 40 mM (lane 4), 60 mM (lane 5), 80 mM (lane 6), 100 mM (lane 7), 120 mM (lane 8), 140 mM (lane 9), 160 mM (lane 10), 180 mM (lane 11), and 200 mM (lane 12). Distilled water was used as the control (0 mM). Suspensions were separated into the supernatant and precipitate, and the supernatant was subjected to SDS-PAGE (10% polyacrylamide). (B) The proportion of residual canavalin in the supernatant was estimated from the band intensity using ImageJ. Data are expressed as the means ± standard deviations of three independent replicates.

The NaCl concentration-dependency of canavalin solubilization was analyzed by suspending MgCl_2_-precipitated canavalin (Figure 2A, lane 1) in various concentrations of NaCl (range: 0–200 mM, Figure 2). The mixtures were then separated into supernatants and precipitates (Figure 2A, lanes 2-12). The intensity of the canavalin bands indicated the amount of canavalin present (Figure 2B). Soluble canavalin gradually increased with increasing NaCl concentrations until it reached a maximum (102.5 ± 7.9%) in the presence of 180 mM NaCl. These results indicated that the solubility of MgCl_2_-precipitated canavalin was NaCl concentration-dependent and that the precipitated canavalin was almost entirely solubilized at high concentrations of NaCl.

### 3.3 Structural differences between soluble canavalin in sword bean extracts and in high concentrations of NaCl

As previously described, soluble canavalin in sword bean extracts can be maintained in a soluble form via the addition of low concentrations of NaCl (Nishizawa and Arii, 2016). However, as shown in Figure 2, almost half of the MgCl_2_-precipitated canavalin was insolubilized in the presence of NaCl at concentrations <60 mM. This inconsistency implies that although canavalin in sword bean extracts and in solutions with high concentrations of NaCl have similar soluble forms, their structures differ.

**Figure 3:**
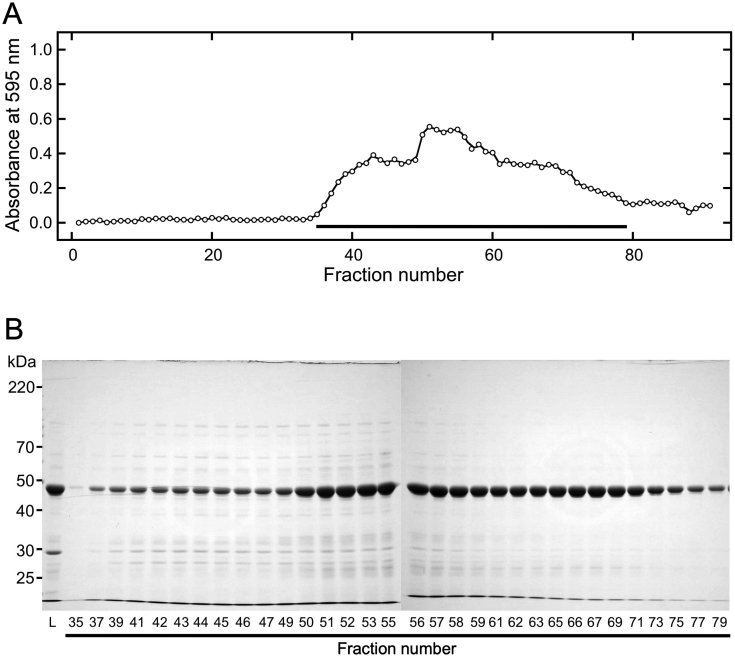
Gel filtration chromatography of proteins extracted from sword beans. Extracted proteins in distilled water (2 mL) were loaded onto a gel filtration column that was equilibrated with distilled water at a flow rate of 0.5 mL/min. Eluted samples were collected in volumes of 1.5 mL. (A) After gel filtration chromatography, each eluted sample was reacted with Bradford dye reagent and then measured at 595 nm. Black bars indicate fractions analyzed by SDS-PAGE. (B) The loaded (L) and eluted samples were subjected to SDS-PAGE (10% polyacrylamide). Numbers represent fraction numbers in panel A. Eluted samples were distinguished between fraction numbers 35–55 and 56–79 on different gel plates.

**Figure 4:**
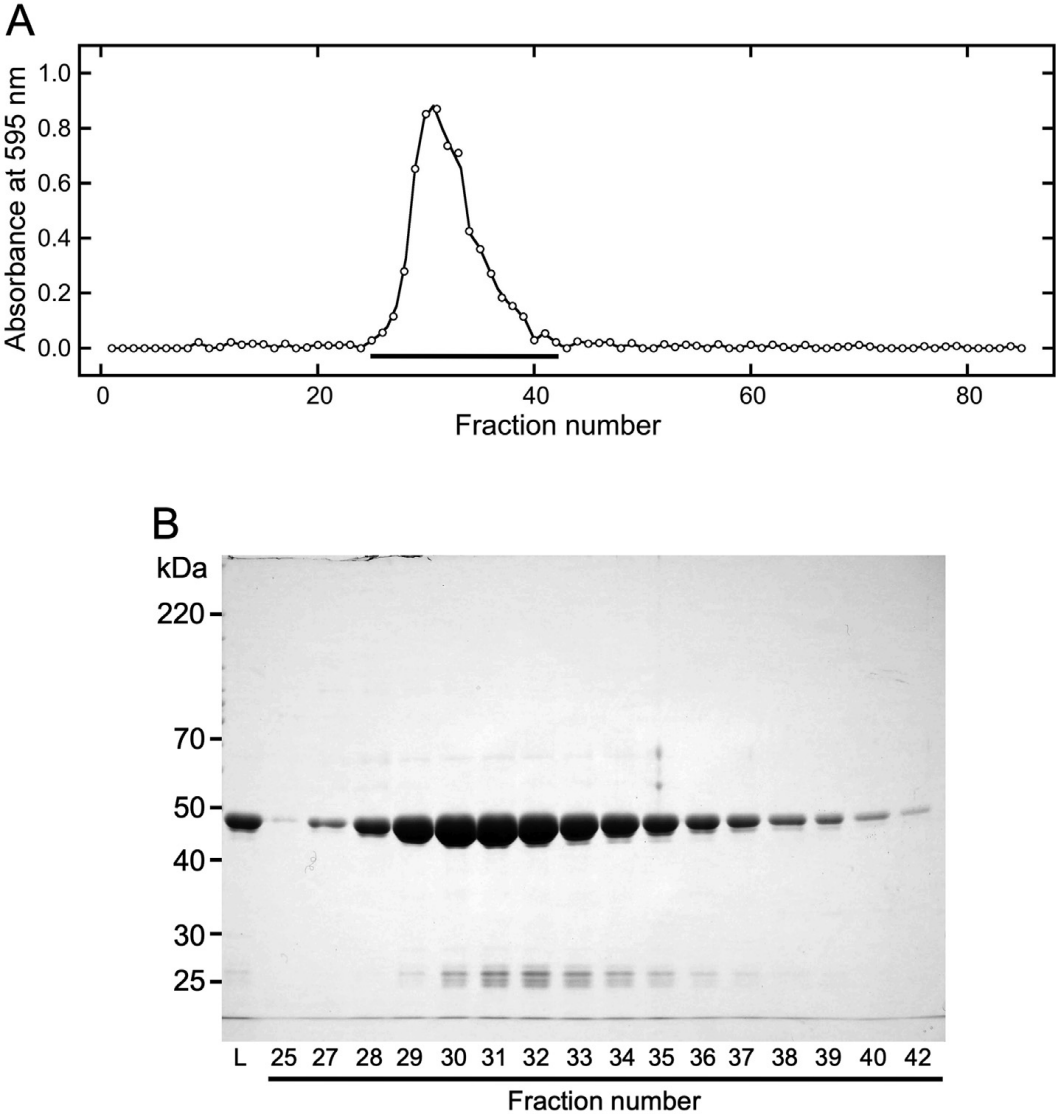
Gel filtration chromatography of proteins in the suspension with NaCl. Canavalin in the sword bean extract was precipitated via the addition of MgCl_2_ at a final concentration of 15 mM. Precipitated canavalin was suspended in 200 mM NaCl, and the suspension was separated into the supernatant and precipitate by centrifugation. Supernatant proteins (2 mL) were loaded onto a gel filtration column that was equilibrated with 200 mM NaCl at a flow rate of 0.5 mL/min. Eluted samples were collected in volumes of 1.5 mL. (A) The eluted samples were reacted with Bradford dye reagent and then measured at 595 nm. Black bars indicate fractions analyzed by SDS-PAGE. (B) The loaded (L) and eluted samples were subjected to SDS-PAGE (10% polyacrylamide). Numbers represent fraction numbers in panel A.

To detect these structural differences, soluble canavalin in sword bean extract (Figure 3) and in a high-concentration NaCl solution (Figure 4) was analyzed by gel filtration chromatography. Proteins in the crude extract were clearly eluted from fraction numbers 35 to 80 (Figure 3A). The abundance of eluted proteins was observed to increase in two primary stages. In the first stage, the abundance (equivalent to absorbance) was seen to steadily increase beginning at fraction 35 through to fraction 43, at which point it plateaued until fraction 49. In the second stage, protein abundance consistently increased from fraction 49 to 52 and plateaued from 52 to 55 after which the protein abundance was seen to steadily decrease until fraction 80. The peak abundance was determined to occur in fraction 51. SDS-PAGE analysis indicated that the molecular weight of the major eluted protein was approximately 47 kDa (Figure 3B). The theoretical molecular weight of canavalin is ~47.6 kDa (Sumner et al., 1983). These results indicate that a large proportion of the eluted proteins comprised canavalin. The molecular weight standard was also eluted under the same conditions. According to an estimate based on the elution pattern of the molecular weight standard, proteins with this molecular weight are theoretically eluted at fraction number 39. As shown in the elution pattern and SDS-PAGE analysis, the elution position of canavalin was broad, although it was also eluted at fraction number 39. These results indicate that the canavalin in sword bean extract was eluted in a position that corresponds to a lower molecular weight monomeric form. Hence, from the elution position, it was deduced that canavalin existed as a monomer and in an unstable form within the crude extract.

In the high-concentration solution, proteins were eluted primarily from fraction 25 to 39 (Figure 4A). The peak abundance was determined to occur in fraction 31. Moreover, SDS-PAGE analysis indicated that the majority of eluted proteins were canavalin (Figure 4B). In previous studies, crystal structures of canavalin indicated that canavalin occurred in its homo-trimer form (McPherson, 1980; Ko et al., 2001). The hypothetical molecular weight of trimeric canavalin is 142.8 kDa and proteins of this size are theoretically eluted in fraction number 32. Although fraction number 31 was estimated to be have a molecular weight of 178.1 kDa, the elution position indicated that the MgCl_2_-precipitated canavalin suspended in 200 mM NaCl was in its trimer form. In addition, the results presented in Figures 3 and 4 indicate that the canavalin in the sword bean extract was structurally different from that in the 200 mM NaCl solution, which was prepared by solubilizing MgCl_2_-precipitated canavalin.

**Figure 5:**
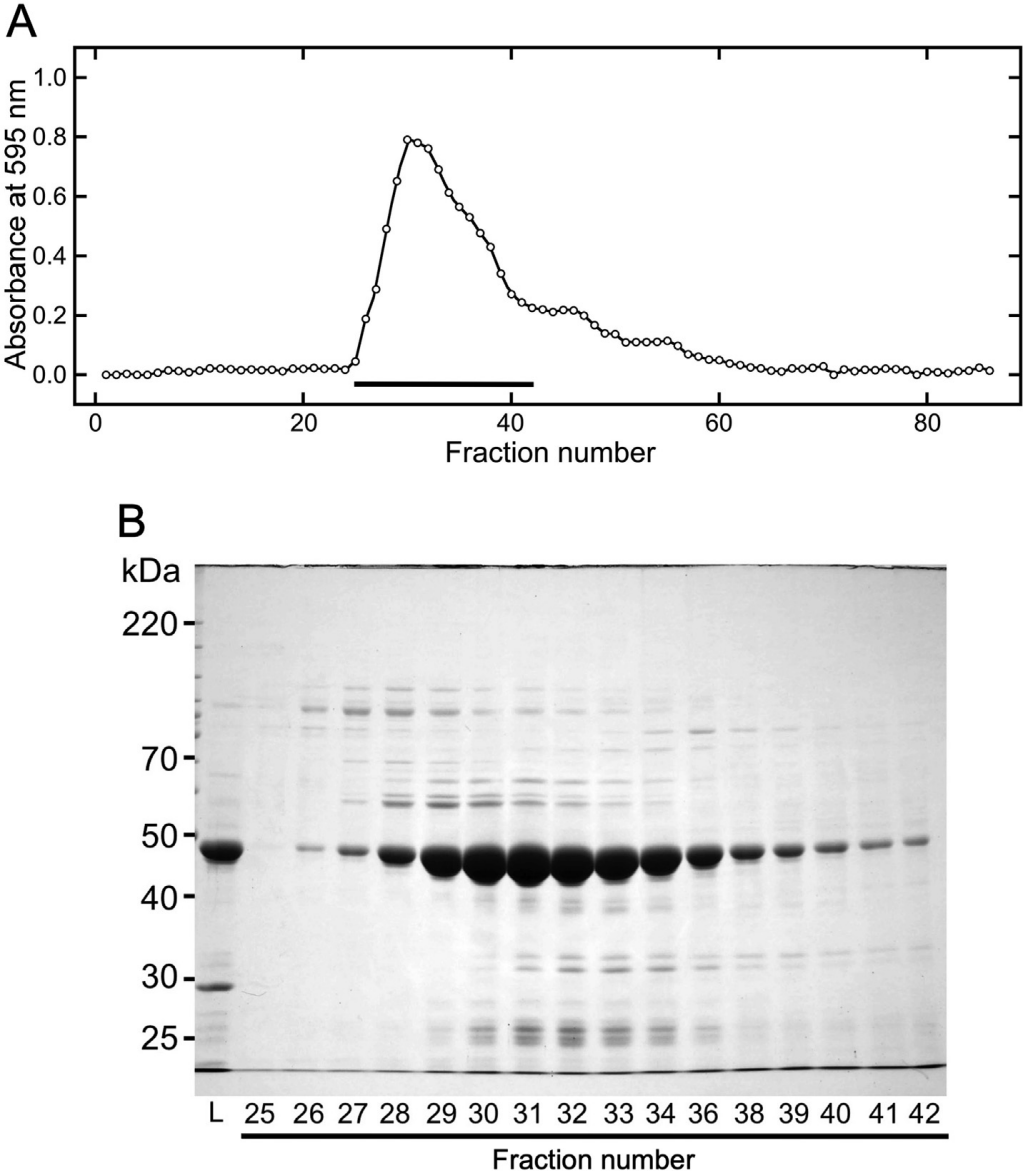
Gel filtration chromatography of proteins in the sword bean extract and NaCl mixture. NaCl was added to the sword bean extract at a final concentration of 200 mM. The NaCl mixture was separated into the supernatant and precipitate by centrifugation. Supernatant proteins (2 mL) were loaded onto a gel filtration column that was equilibrated with 200 mM NaCl at a flow rate of 0.5 mL/min. Eluted samples were collected in volumes of 1.5 mL. (A) Each eluted sample was reacted with Bradford dye reagent and then measured at 595 nm. Black bars indicate fractions analyzed by SDS-PAGE. (B) The loaded (L) and eluted samples were subjected to SDS-PAGE (10% polyacrylamide). Numbers represent fraction numbers in panel A.

As shown in a previous study by Nishizawa and Arii (2016), soluble canavalin in the sword bean extract remained in a soluble form via the addition of NaCl. To investigate the effect of directly changing the NaCl concentration on the quaternary structure of canavalin, a sample was prepared by adding NaCl to the sword bean extract at a final concentration of 200 mM. Proteins in the sample were then separated by gel filtration chromatography (Figure 5). Eluted samples were analyzed in the same way as previously described (Figure 5A). The abundances of eluted proteins were dramatically increased in fraction number 25, with peak abundance measured in fraction 30. The pattern of protein abundance was similar to that observed for soluble canavalin prepared from MgCl_2_-precipitated canavalin. However, the decrease in eluted proteins was comparatively more gradual than that observed with the soluble form prepared from MgCl_2_-precipitated canavalin. SDS-PAGE analysis indicated that the main eluted protein was canavalin and that similar protein concentrations were eluted in fractions 30 to 32 (Figure 5B). These results indicated that canavalin underwent alterations in its structure to form a trimer following the addition of high concentrations of NaCl.

### 3.4 Elution pattern of canavalin in the presence of high-concentration MgCl_2_

**Figure 6:**
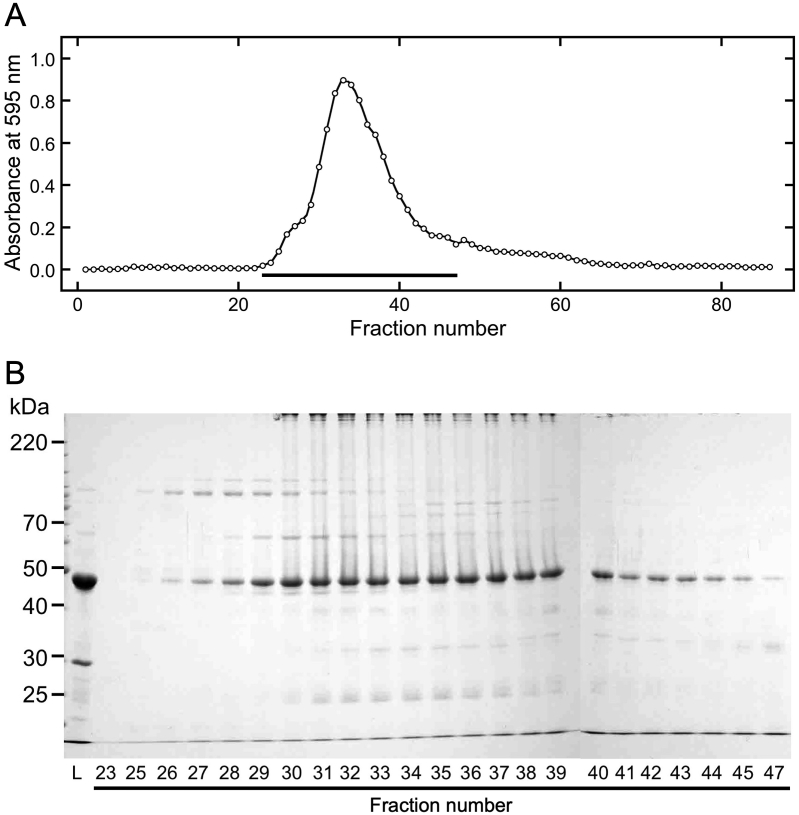
Gel filtration chromatography of proteins in the sword bean extract and MgCl_2_ mixture. MgCl_2_ was added to the sword bean extract at a final concentration of 60 mM. The MgCl_2_ mixture was separated into the supernatant and precipitate by centrifugation. Supernatant proteins (2 mL) were loaded onto a gel filtration column that was equilibrated with 60 mM MgCl_2_ at a flow rate of 0.5 mL/min. Eluted samples were collected in volumes of 1.5 mL. (A) The eluted samples were reacted with Bradford dye reagent, and then measured at 595 nm. Black bars indicate fractions analyzed by SDS-PAGE. (B) The loaded (L) and eluted samples were subjected to SDS-PAGE (10% polyacrylamide). Numbers represent fraction numbers in panel A. Eluted samples were distinguished between fraction numbers 23–39 and 40–47 on different gel plates.

To investigate the effects of MgCl_2_ on the quaternary structure of canavalin, it was added to the sword bean extract at a final concentration of 60 mM, at which canavalin was in a soluble form. Supernatant proteins were separated by gel filtration chromatography (Figure 6). Eluted proteins increased in abundance beginning at fraction 24 with the peak abundance measured in fraction number 33. The elution pattern strongly suggested that the soluble canavalin that was prepared from sword bean extract with the addition of MgCl_2_ was also in a trimer form. SDS-PAGE analysis indicated that the primary eluted protein was canavalin (Figure 6B). However, the abundance of eluted canavalin was much lower than that eluted in distilled water and 200 mM NaCl. Furthermore, the resulting molecular weights of eluted proteins (>220 kDa) suggested that proteins formed aggregates in the samples from fraction 30 to 39. These results indicate that canavalin might more readily aggregate in the presence of MgCl_2_ compared to that in NaCl (Figures 4 and 5).

**Figure 7:**
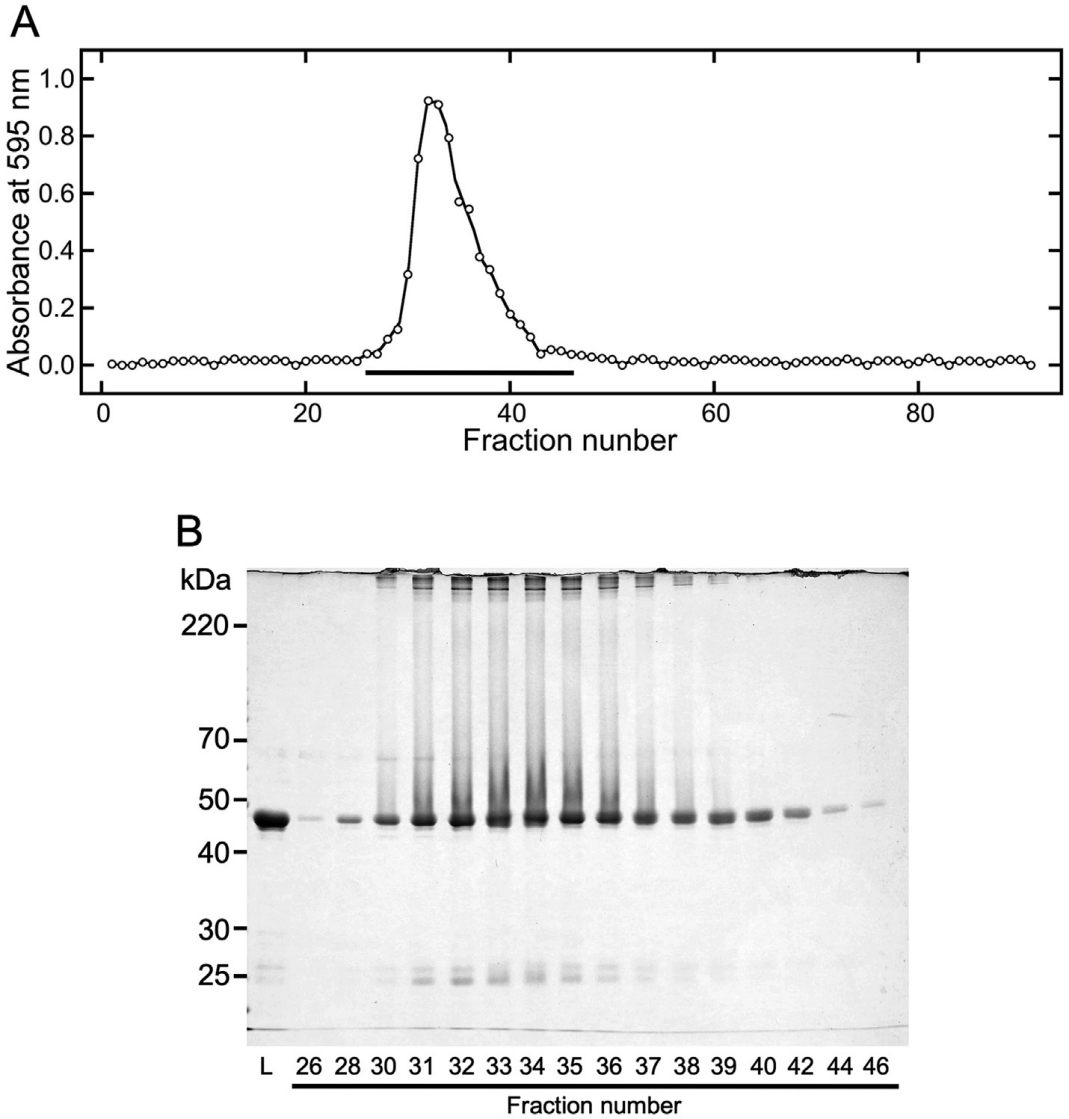
Gel filtration chromatography of proteins in the suspension with MgCl_2_. Canavalin in the sword bean extract was precipitated via the addition of MgCl_2_ at a final concentration of 15 mM. Precipitated canavalin was suspended in 60 mM MgCl_2_, and the suspension was separated into the supernatant and precipitate by centrifugation. Supernatant proteins (2 mL) were loaded onto a gel filtration column that was equilibrated with 60 mM MgCl_2_ at a flow rate of 0.5 mL/min. Eluted samples were collected in volumes of 1.5 mL. (A) The eluted samples were reacted with Bradford dye reagent and then measured at 595 nm. Black bars indicate fractions analyzed by SDS-PAGE. (B) The loaded (L) and eluted samples were subjected to SDS-PAGE (10% polyacrylamide). Numbers represent fraction numbers in panel A.

As described in a previous study by Nishizawa and Arii (2016), MgCl_2_-precipitated canavalin can be solubilized in 60 mM MgCl_2_. To investigate the effect of MgCl_2_ on the quaternary structure of canavalin, resolubilized canavalin was prepared by solubilizing the MgCl_2_-precipitated canavalin in 60 mM MgCl_2_. Then, supernatant proteins were separated by gel filtration chromatography (Figure 7) and eluted samples were analyzed as described previously (Figure 7A). The abundance of eluted proteins increased beginning in fraction 26 and the peak abundance was observed in fraction 32. These results indicated that the soluble canavalin prepared by adding MgCl_2_ to the MgCl_2_-precipitated canavalin also existed as a trimer. Eluted samples were subjected to SDS-PAGE (Figure 7B) and the main eluted protein was canavalin. However, as also seen in Figure 6, the amount of eluted canavalin was much lower than that after elution in distilled water and 200 mM NaCl; moreover, proteins in the sample formed aggregates in fractions 30 to 39. These results confirm that canavalin more readily forms aggregates in the presence of MgCl_2_ compared to that in solutions with NaCl (Figures 4 and 5).

## 4 Discussion

In this study, we found that the quaternary structure of soluble canavalin in sword bean extract differed with high concentrations of salts. This is the first study, to our knowledge, to describe canavalin as a soluble and unstable monomer in sword bean crude extract. Furthermore, this is the first report to show that the quaternary structure of canavalin changes from a monomer to a trimer in the presence of high concentrations of NaCl (Figure 4) and MgCl_2_ (Figure 6). The ionic strength of 200 mM NaCl (0.20) is similar to that of 60 mM MgCl_2_ (0.18), implying that the trimer form is induced by changing the ionic strength. This result indicates that increasing ionic strength is an important factor for trimer formation. In addition, it is known that globulins are insoluble in deionized water but dissolve in dilute salt solutions. This evidence indicates the possibility that the specific property of globulins is derived from the ion strength-dependent alterations in quaternary structure.

Interestingly, the soluble trimer form of canavalin slowly became aggregated in the presence of 60 mM MgCl_2_ (Figures 6 and 7). This aggregation was also observed when 200 mM CaCl_2_ was added in our preliminary experiment (data not shown). However, in the presence of 200 mM NaCl, aggregation was not observed (Figures 4 and 5). Previous studies have reported that MgCl_2_ can precipitate canavalin in the extract; however, NaCl does not have the same effect (Nishizawa and Arii, 2016). In a similar process involving the formation of tofu-like precipitates salt bridges formed via divalent cations trigger the aggregation of soybean proteins (Arii and Nishizawa, 2018), which are also classified in the globulin class, along with canavalin. Hence, the different properties observed with canavalin following the addition of different salts might also be induced by the formation of salt bridges through divalent cations triggering the aggregation of the trimer form of canavalin in the presence of MgCl_2_.

A previous study reported that MgCl_2_-precipitated canavalin can become solubilized in the presence of high concentrations of salts but not in distilled water (Nishizawa and Arii, 2016). These phenomena raise the question of why the monomeric form is soluble in extract. The extract would contain specific minerals, as estimated by Mohan and Janardhanan (1994), to be approximately 0.32 mM sodium, 63 mM potassium, 73 mM calcium, and 3.4 mM magnesium. However, since the extract was prepared after dried beans had been soaked in 10 volumes of distilled water for 18 h, the true concentrations of these minerals would be much lower than that observed in the previous study. In addition, if these minerals contributed to the solubilization of canavalin in the extract, canavalin would be in the trimer form within the extract. Minerals present with the beans would not contribute to the solubilization of canavalin in the extract. In addition, the trimer form of canavalin might become dissociated in distilled water to the unstable monomer form. However, since the MgCl_2_-precipitated canavalin would be more stable than the soluble monomer form, the MgCl_2_-precipitated canavalin would not be solubilized in distilled water. To understand the relationship between structure and solubility, it would be important to compare the secondary structure of the monomer with that of the trimer. However, it is difficult to isolate the monomer because it immediately changes to a trimer or precipitated form under various conditions. For this comparison, we must find the condition that can keep canavalin in its monomeric form.

Canavalin was the first protein to be isolated from jack beans (Sumner, 1919), and its primary structure was later characterized in both the sword bean (Yamauchi et al., 1988; Takei et al., 1989) and jack bean (Ng et al., 1992, 1993) using cDNA sequencing. In previous studies, jack bean canavalin was purified and crystalized in the trimer form in the presence of high concentrations of NaCl (Ko et al., 1993, 2000; Ng et al., 1993). Although three-dimensional structures of canavalin from sword beans have not yet been determined, the primary canavalin structures from both species differ by only 2 of the 419 amino acids; they would, therefore, be expected to have a high homology in their three-dimensional structures as well. These reports were consistent with the findings of the present study that canavalin exists in a trimer form in the presence of higher concentrations of NaCl (Figure 4) or MgCl_2_ (Figure 6).

Canavalin is classified as a vicilin from the 7S globulin family. The three-dimensional structure of β-conglycinin, which is classified in the same group, also has a trimer structure (Maruyama et al., 2001). β-conglycinin was also purified and crystallized in the presence of high concentrations of NaCl (Maruyama et al., 2001; Morita et al., 1996). These findings suggest that 7S globulins occur in trimeric form in the presence of high concentrations of NaCl. However, it remains unclear whether 7S globulins are present as trimers in bean seeds. As shown in Figure 3, sword bean canavalin in the extract was found to be in the monomer not trimer form. This suggests that canavalin in sword bean seeds is present in the monomer form. Therefore, β-conglycinin in seeds might also be present in monomer form. As reported in our previous study, MgCl_2_-precipitated canavalin was not solubilized in distilled water (Nishizawa and Arii, 2016). In the crude extract, canavalin occurs as a soluble monomer; however, the conditions that enable the occurrence of this form were not elucidated in the present study, and thus require further investigation.

## 5 Conclusions

In conclusion, we found that sword bean-extracted canavalin in distilled water occurs as a monomer structure; however, the addition of high concentrations of NaCl or MgCl_2_ induces a change from the monomeric to the trimeric form. In future studies, we aim to further investigate the conditions that enable the existence of soluble monomers. Our data serve as an important reference to analyze sword bean protein characteristics and 7S globulin characteristics.

## Declarations

### Author contribution statement

Kaho Nishizawa: Performed the experiments; Analyzed and interpreted the data; Contributed reagents, materials, analysis tools or data; Wrote the paper.

Yasuhiro Arii: Conceived and designed the experiments; Performed the experiments; Analyzed and interpreted the data; Contributed reagents, materials, analysis tools or data.

### Funding statement

This work was supported by JSPS KAKENHI (grant number JP18K14429).

### Competing interest statement

The authors declare no conflict of interest.

### Additional information

No additional information is available for this paper.
